# The Field of Science

**Published:** 1894-03-17

**Authors:** 


### The Field of Science.

The revelations which have lately come to light on the subject of London bakeries are of a more serious nature than had been expected. Dr. Waldo, who has made this matter a subject of grave consideration, lectured at the Sanitary Institute on the results of his labours. Bread, besides being an article of universal necessity, is peculiarly liable, as we all know, to absorb moisture and gaseous substances; yet, as the lecturer pointed out, at least half of the total output of this staple article of food proceeds from "cellar bakeries." These cellar bakeries sound little better in description than they are in reality; they are for the most part damp, low basement rooms, with dark walls, unceiled roofings, and rough and uneven floors. As to ventilation, that is conspicuous by its absence; neither is daylight included in the programme, the cellars being lighted by flaring gas-jets instead.

"It will be seen, therefore," in Dr. Waldo's words, 'that not only is the air systematically poisoned, from within, but also no provision is made for that constant renewal from without which is essential to health. We can safely assert that no man could habitually work in an atmosphere even approaching that which has been portrayed without sooner or later sustaining serious injury to his health." Our authority refers us to Dr. Ogle's tables of comparative mortality of males between twenty-five and sixty-five years of age, founded upon the death registers of 1880-1-2, which offer, as he remarks, some very suggestive figures on this head. From them we learn that in one hundred different occupations bakers occupy the following relative proportions: In suicide they come third, in alcoholism seventh, in liver disease eleventh, in diseases of the circulatory system eleventh, and twelfth in diseases of the nervous system. It seems high time a reform should be suggested for these existing evils, and though Dr. Waldo does not stand alone in pointing out the pernicious effect of these underground cellars not alone on the article there manufactured, but on all concerned, yet it is due to him that he has exposed in tangible form the magnitude of the existing evil.

